# Analysis of the TCR Repertoire in HIV-Exposed but Uninfected Infants

**DOI:** 10.1038/s41598-019-48434-4

**Published:** 2019-08-16

**Authors:** Benjamin Gabriel, Carey Medin, Jeremiah Alves, Ruth Nduati, Rose Kerubo Bosire, Dalton Wamalwa, Carey Farquhar, Grace John-Stewart, Barbara L. Lohman-Payne

**Affiliations:** 1 Institute for Immunology and Informatics, Department of Cell and Molecular Biology, University of Rhode Island, Providence, RI 02903 USA; 2 Department of Paediatrics and Child Health, University of Nairobi, Nairobi, 30197 Kenya; 3 Department of Medical Epidemiology and Biostatistics, Karolinska Institute, Stockholm, 17177 Sweden; 4 Department of Global Health, University of Washington, Seattle, Washington 98104 USA; 5 Departments of Medicine, University of Washington, Seattle, Washington 98104 USA; 6 Departments of Epidemiology, University of Washington, Seattle, Washington 98104 USA; 7 Departments of Pediatrics, University of Washington, Seattle, Washington 98104 USA

**Keywords:** Next-generation sequencing, Immunology

## Abstract

Maternal human immunodeficiency virus (HIV) infection has been shown to leave profound and lasting impacts on the HIV-exposed uninfected (HEU) infant, including increased mortality and morbidity, immunological changes, and developmental delays compared to their HIV-unexposed (HU) counterparts. Exposure to HIV or antiretroviral therapy may influence immune development, which could increase morbidity and mortality. However, a direct link between the increased mortality and morbidity and the infant’s immune system has not been identified. To provide a global picture of the neonatal T cell repertoire in HEU versus HU infants, the diversity of the T cell receptor beta chain (TRB) expressed in cord blood samples from HEU infants was determined using next-generation sequencing and compared to healthy (HU) infants collected from the same community. While the TRB repertoire of HU infants was broadly diverse, in line with the expected idea of a naïve T cell repertoire, samples of HEU infants showed a significantly reduced TRB diversity. This study is the first to demonstrate differences in TRB diversity between HEU and HU cord blood samples and provides evidence that maternal HIV, in the absence of transmission, influences the adaptive immune system of the unborn child.

## Introduction

Since the introduction of programs to prevent mother-to-child transmission (PMTCT) of HIV in 2000, the population of HIV-uninfected infants born to HIV-infected mothers is growing. Between the years 2009 and 2014 this group of children, known as HIV-exposed uninfected (HEU) infants, increased by more than 1 million^1^. In countries with high HIV prevalence up to 30–40% of births will be to HIV-positive mothers^2^, while HIV transmission rates during pregnancy have been reduced to less than 1% in resource-rich countries with high coverage of PMTCT^3,4^.

There is emerging evidence that HEU infants differ from HIV-unexposed (HU) infants. HEU have a higher risk of morbidity, mortality, growth compromise, developmental delays, and differences in immune development^5–14^. Immunological differences have been identified mainly at the cell population level. HEU infants have lower CD4 T cell counts compared to HU infants, and these lower T cell counts correlate inversely with viral loads measured in antiretroviral therapy-(ART)-treated women at the time of delivery^7,13^. Additional alterations found in T cell populations in HEU infants compared to HU infants include: (a) increased frequencies of activated T cells during early lifetime, (b) increased memory T cell populations, and (c) increased populations of immature T cells, which indicates a disturbed T cell development^6^. Further evidence of a defective development of various areas of the immune system can be found at the level of innate immunity, such as monocytes^15^ or natural killer (NK) cells^16^. Some immunological changes may reflect exposure to antiretroviral therapy, such as lower neutrophil counts found in HEU infants compared to HU infants^8,17^.

The significance of immune changes in HEU children is undefined. Reports of vaccine-induced cellular immune and antibody responses in HEU infants are contradictory: vaccines against Bacille Calmette-Guérin (BCG) and pertussis stimulate lower levels of response in HEU than HU, while others report a full vaccine schedule for pertussis and pneumococcus results in similar levels of response among HEU and HU infants^18,19^.

Our study is based on the hypothesis that *in utero* exposure to HIV interacts with the development of the immune system of the unborn child on a broad scale. To test this assumption, the T cell repertoire of the newborn was examined using next-generation sequencing of umbilical cord blood, which represents the status of prenatal immune exposure, and the time point of the most naïve immune system. We have designed a study drawing on birth samples from two historic cohorts, one with *in utero* exposure to HIV that did not lead to infection, and one with no exposure to HIV. We further selected infant samples from the larger parent studies with the lowest probability of dysregulation: Infants born at normal birth weight and gestational age from mothers with no history of sexually transmitted diseases other than HIV.

HEU specimens used in this study (n = 19) were collected between 1992 and 1997 in Nairobi, Kenya^20^. Of particular importance here is the absence of any antiretroviral medication given to the mothers for PMTCT, which was only introduced in the 2000s. By drawing on samples from the pre-ART era in this region, we can rule out any drug effect on the T cell repertoire of HEU infants, something not possible within contemporary studies.

An HIV-uninfected birth cohort was established between 2003 and 2005^21^, which served as the source for the control samples used in this study (n = 9). Participants of this cohort were enrolled in the same geographical region (Nairobi, Kenya) and characterized by similar socio-economic factors. The careful consideration regarding sample selection allows for the comparability of the two sample sources.

## Results

### Health-related assessment of infants and immune status of mothers at birth

**Figure 1:**
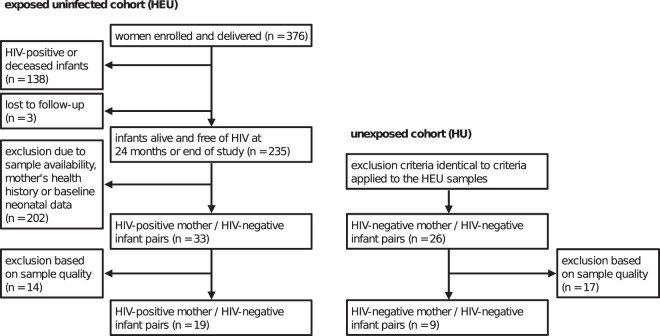
Schematic representation of study design and sample selection. The work presented here is based on HEU and HU cord blood samples of two studies conducted in Nairobi, Kenya^20,21^. Sample availability (presence of mother-child sample pairs), health history of the mother (no maternal history of *Treponema pallidium*, *Trichomonas vaginallis*, *Candida*, *Neisseria gonorrhea*, or *Chlamydia*), as well as baseline neonatal data were used as selection criteria. Both studies share similar socio-economic determinants and health-related information, allowing comparability of the samples.

Health-relevant data on mother-infant pairs were collected within a period of up to 2 years for HEU infants, 6 months for HU infants, and their mothers (Fig. 1). Specifically, review of the health data allowed for selection of samples from particularly healthy mothers and infants. As a result, HEU and HU samples showed no significant differences in baseline birth anthropromorphic data collected (Table 1).

**Table 1:** Health status of infants after birth.

	HIV-exposed uninfected (HEU)	HIV-unexposed (HU)
Gestational age	39.7 weeks (IQR 37.7–41.0)	^†^
Birth weight	3.3 kg (IQR 3.0–3.5)	3.1 kg (IQR 2.9–3.5)
Birth length	49.0 cm (IQR 47.0–50.0)	47.0 cm (IQR 46.0–50.0)
Head circumference	36.0 cm (IQR 35.0–36.0)	34.7 cm (IQR 34.0–35.5)
Apgar score	10.0 (IQR 10–10)	10.0 (IQR 10–10)
Dubowitz score	57.0 (IQR 49.5–62.0)	^‡^

Gestational age was determined using the Dubowitz score and reflects the theoretical gestational age (0.2642× Dubowitz score + 24.595). ^†^Data not available; ^‡^not measured.

In addition to routinely collected health data of HEU and HU mother-infant pairs, HIV-relevant data were collected for the group of HIV-positive mothers. HIV-positive mothers included in this study were characterized as moderately immunosuppressed with a median CD4 T cell count of 422 cells/ml (interquartile range, IQR 316–537) and a CD4: CD8 ratio of 0.574 (IQR 0.321–0.723) for pre-delivery time points (31–38 weeks of pregnancy). The median viral load at delivery for this subgroup of samples was 4.2 log_10_ RNA copies/ml plasma (range of 1.7–5.5 log_10_ RNA copies/ml plasma).

### Comparison of sequencing performance and assembled clonotypes between samples of HIV-exposed uninfected and unexposed infants

We first evaluated the quality of the extracted samples and the resulting sequence data. Sequencing of the TCR repertoire of HEU and HU cord blood samples was performed using an amplicon-based library preparation and sequencing on Illumina’s MiSeq instrument. The sequencing performance, as measured by raw read counts, was within the expected range of 1 million reads for each sample. Sequencing samples of HEU cord blood achieved nearly equal number of raw reads than HU cord blood samples: median raw read counts for HEU and HU cord blood samples were 9.55 × 10^5^ (IQR 8.32–10.81 × 10^5^) vs 7.26 × 10^5^ (IQR 6.5–9.22 × 10^5^), respectively. Despite similar raw read counts, the number of successfully identified TRB sequences differed substantially between the two sample groups. The number of successfully assigned TRB sequences within HEU samples was 6.84 × 10^5^ (IQR 5.08–9.07 × 10^5^) compared to 0.72 × 10^5^ (IQR 0.57–1.68 × 10^5^) for HU samples. Based on the assigned TRB sequences we were able to identify a median 17,242 (IQR 12,290–33,709) unique clonotypes in HEU samples and a median of 505 (IQR 426–1,374) unique clonotypes in HU samples. Each clonotype represents a defined TRB-CDR3 (complementarity determining region 3) sequence combination. To investigate the low number of assigned sequences and unique clonotypes in the HU samples, we evaluated the quality of the extracted RNA. We observed differences between the two groups as measured by RNA integrity numbers (RIN). Lower RIN scores were measured for the samples of the HU group (median 2.5, IQR 2.1–2.7) compared to the samples of the HEU group (median 7.9, IQR 7.2–8.6), potentially causing the loss of successfully assigned TRB sequences. Taken together, samples from HU infants yielded fewer TRB sequences and identifiable clonotypes than samples from HEU infants due to lower RIN scores.

To assess the effect of the measured RIN scores on the sequencing results, we compared the usage of the variable region of the T cell receptor beta chain gene (TRBV) across both HEU and HU groups. We observed no significant difference in the proportions of common TRBV families detected in the HEU compared to the HU population. In addition, the relative proportions were similar to those identified as highly expressed TRBV families within samples from another study^22^, available at the Sequence Read Archive (SRA). This observation suggests that lower RIN scores in HU samples led to lower reads, but did not influence the expression pattern of the TRBV families to each other and thus did not introduce any bias of the TRB diversity measurements.

### Diversity calculation of top 200 clonotypes

The diversity of samples with respect to their TRB repertoire is largely driven by expanded clonotypes, which can be found at the top of ordered frequency tables of TRB clones. As the focus of this study was to investigate the occurrence of expanded clonotypes within cord blood samples of HEU and HU infants, the analysis was limited to the first 200 clonotypes. Each of the cord blood samples was represented by a list of the 200 most frequently detected TRB sequences, for which the TRB proportions were recalculated and used to determine the diversity scores.

**Figure 2:**
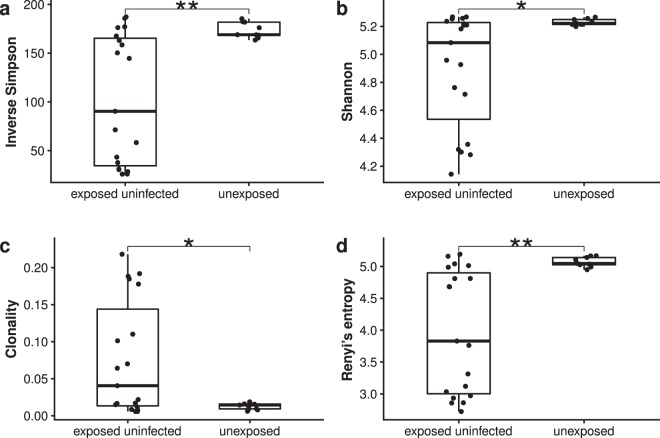
Diversity indices calculated for TRBV genes from HEU (n = 19) and HU (n = 9) cord blood samples. Inverse Simpson diversity (**a**) Shannon diversity (**b**) clonality (**c**) and Rényi’s entropy for *α* = 3 (**d**) were calculated using the top 200 clonotypes for each cord blood sample. Statistical testing was done using Wilcoxon rank-sum test. Significant levels are given as *<0.05, **<0.01, and ***<0.001.

Simpson and Shannon diversity, which differ in the weighting of high abundance clonotypes, are widely used indices for estimating diversity. For the group of HEU samples, the median inverse Simpson diversity score was significantly lower: 90.36 (IQR 34.46–165.31) compared to the HU group median diversity score of 168.89 (IQR 168.39–181.78, *p* 0.0073, Fig. 2a). This suggests clonal expansions within the samples of the HEU infant group, which lead to a reduction in clonal diversity and thus diversity scores. Interestingly, HU infants showed a similar maximum inverse Simpson diversity score of 185.35 compared to 187.19 for the HEU group, indicating a similar maximum diverse TRB repertoire.

Median Shannon diversity scores for HEU and HU cord blood samples were 5.08 (IQR 4.36–5.24) for the HEU group and 5.22 (IQR 5.21–5.25) for the HU group (*p* 0.0477, Fig. 2b). Although there is less difference between the median Shannon diversity indices of the HEU group and the HU group, the variance of the indices of the HEU group (*σ*^2^ = 0.1692) was substantially greater compared to the variance of the Shannon diversity indices of the HU group (*σ*^2^ = 0.0005).

Clonality, which is based on Pielou’s evenness, and Rényi’s entropy are additional diversity measures used in different disciplines, including the determination of T and B cell receptor (TCR/BCR) diversity. Clonality values for HEU cord blood samples ranged between 0.006 and 0.218, with a median clonality value of 0.041 (Fig. 2c). This again represents a diversity measure with a much greater variance compared to the HU sample group, for which a much smaller range of clonality values was determined (0.006–0.019, median of 0.014, *p* 0.0477).

**Figure 3:**
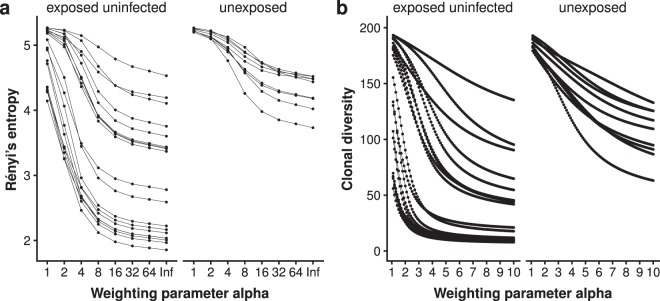
Diversity profiles for cord blood samples from HIV-exposed uninfected (n = 19) and HIV-unexposed (n = 9) infants. Rényi’s entropy (**a**) and clonal diversity (**b**) were plotted in a range of *α* = 1 to *α* = ∞ or *α* = 10, respectively.

Rényi’s entropy (Fig. 2d), as well as clonal diversity, can be calculated for different alpha values which changes the weighting of high abundance clonotypes within the top 200 clonotype tables. Figure 3 shows Rényi’s entropy for *α* = 1 to *α* = ∞ and clonal diversity for *α* = 1 to *α* = 10. Increasing the *α* value for both Rényi’s entropy and clonal diversity causes the two groups to drift apart, with the diversity values of the HEU group decreasing significantly faster than the diversity values of the HU group. With a weighting factor *α* of 3, the median Renyi entropy between the two groups showed a difference of 1.216 points and a *p*-value of 0.001 (Fig. 2d).

In summary, our results for TRBV diversity in HU compared to HEU samples demonstrate that (1) HU cord blood diversity estimates are near maximal and (2) in the presence of a reduced likelihood of detecting a difference in diversity, all diversity measurements presented here showed significant differences between the T cell diversity of HEU and HU umbilical cord blood samples, with a lower diversity detected in a subset of HEU infants. This reduced diversity is associated with a greater number of high abundance clonotypes, consistent with clonal expansions.

### Rank-abundance plotting

**Figure 4:**
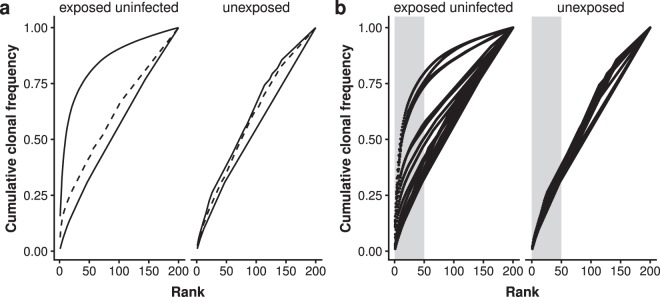
Rank abundance plotting of top 200 clonotypes. (**a**) Curves representing the median diversity scores (dashed line) for the two groups, HEU (n = 19) and HU (n = 9). Solid lines indicate the maximum and minimum diversity scores of the respective group. (**b**) Curves for the individual samples of the two groups. Grey areas highlight the clonotypes of rank one to 50.

The estimation of diversity involves the risk of an over-simplification of the dataset since diversity indices represent a mathematical transformation of the underlying data into a single value. This oversimplification may result in the loss of important information. Methods such as rank-abundance plotting avoid this risk by using the unmodified dataset for visualization (Fig. 4). Here, the cumulative frequency of the clonotypes is plotted against the rank of the respective clonotype, resulting in a curve starting near the axis of origin and reaching to the maximum rank on the x-axis, or 100% of the selected clonotypes on the y-axis. Figure 4a shows the rank-abundance plot for the median clonotype frequencies of the two groups of this study. The curves for the HEU and HU group intersect at rank 114, which means that samples of the HEU group show a lower diversity (higher frequency of clonal expansion) regarding ranks 1 to 114. After the clone at rank 114, the differences between the two groups are less pronounced.

Looking at the curves of individual samples (Fig. 4b), it is noticeable that not all cord blood samples follow this scheme. Two groups of HEU infants can be identified with regard to the shape of the curves: One group whose profile is similar to that of HU infants and another group with a clearly distinguishable profile, supporting the observations made using the diversity scores.

In addition, Fig. 4b shows how the proportion of expanded clonotypes is distributed among the top 200 clonotypes. Highly expanded clonotypes are mainly found within the first 50 clonotypes of the HEU group, which can be seen by the steep increase of the curves in this area (Fig. 4b, area highlighted in grey).

### Identification of antigen specificity using VDJdb

The predicted antigen specificity of the T cell clonotypes of HEU and HU cord blood samples was analyzed using the VDJdb database^23^. At the time of the query, the database contained 14,943 entries for TRB sequences, which represents 70% of available human TCR sequences within the database. About 90% of the database entries in the query period were for the species cytomegalovirus (CMV), Influenza A, HIV-1, Homo Sapiens and Epstein–Barr virus (EBV).

**Figure 5:**
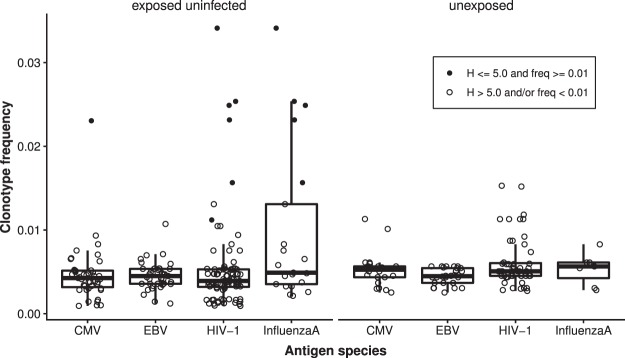
Antigen specificity of the top 200 clonotypes of HEU (n = 19) and HU (n = 9) cord blood samples. Antigen specificity was determined using the VDJdb database and the following search parameters: (1) full matching of CDR3, T cell receptor beta variable and joining genes (TRBV, TRBJ) sequences, (2) VDJdb confidence score of at least 1, and (3) the following number of allowable substitutions (s = 2), indels (id = 1), and total number of mutations (t = 2). Median frequency of clones per group per antigen species are given as box plots. Dot plots are superimposed with the respective individual clonal frequencies. Each dot represents an individual clone. Expanded clonotypes were defined as clonotypes with a minimum frequency of 0.01 within samples showing a Shannon diversity score of 5.0 or less.

T cell clones specific for all pathogens included in the query (CMV, EBV, HIV-1, and influenza-A) were found in umbilical cord blood samples from both groups (Fig. 5). Interestingly, expanded TRB clonotypes in 6 out of 7 HEU cord blood samples with a match in the database were identified as HIV-1-specific. In 5 out of these 6 HEU samples, the expanded TRB clonotypes represent HIV-1/Influenza A cross-reactive T cell clones based on the information stored in the database, while in one HEU sample the expanded clonotype identified was directed against HIV-1 only. HEU samples with expanded HIV-1-specific TRB clonotypes had diversity scores of a median of 4.32 (IQR 4.296–4.458) and showed HIV-1-specific T cell median clone frequencies of 0.024 (IQR 0.021–0.025). The frequency for all pathogen-specific T cell clones within the top 200 clonotypes for each infant group was 0.0041 (IQR 0.0033–0.0052) for HEU and 0.005 (IQR 0.0044–0.0059) for HU.

## Discussion

Using next-generation sequencing technology in combination with a T cell receptor beta chain-targeted library preparation protocol including unique molecular identifier sequences, this study identified significant differences in T cell receptor repertoires between cord blood samples of HEU and HU infants tested by multiple diversity measures and rigorous data quality controls and validation steps. We identified two subsets based on TCR diversity scores within the HEU cord blood samples. One subset was defined by diversity scores comparable to our control group, whereas the other subset was defined by reduced diversity and expanded clonotypes. When identifying the predicted antigen specificities of the top 200 clonotypes, all clones with well above-average elevated frequencies and found exclusively in samples of HEU infants were directed against HIV proteins. This result suggests that the clonal expansion is due to exposure to HIV antigen *in utero*.

Beside HIV-specific T cell clonotypes, T cells specific for other pathogens, specifically cytomegalovirus and influenza virus type A, could be detected in samples from HEU and HU infants. This observation can be made in datasets of other studies, suggesting that these clonotypes are part of the natural T cell receptor repertoire, as these infections are not associated with productive chronic viral replication^22^.

HEU infants show several alterations within their T cell populations after birth, for example reduced naïve CD4/CD8 T cell counts^7^, increased memory CD4 as well as memory and activated CD8 T cell counts and HIV-specific CD4/CD8 T cells as measured by IL-2/IFN*γ* production in response to HIV antigen/peptides^6^. This HIV reactivity was not detected in samples of older HEU infants, concluding that the reactivity gradually wanes over time^6,24^. In addition to this HEU-specific HIV reactivity, stimulation of T cells from HEU infants with antigens of other pathogens such as *Staphylococcus aureus* Cowan show altered immune responses^25^, suggesting a broader impact of the maternal HIV infection to the development of the infants’ immune system. This is of particular importance as there is evidence that these altered immune responses to pathogens impact the ability of the infants to response to vaccinations given during the first months of life^26,27^.

Functional analysis of the expanded T cell clones in cord blood samples of the HEU infants in this study could not be performed because the specimens available were insufficient for *in vitro* cultivation. At the same time, our finding that a subset of HEU cord blood samples showed a broad TRB repertoire suggests that expanded HIV-specific T cell clonotypes were not required for the prevention of HIV transmission from ART-naïve mothers to their children. It is known that maternal viral load is a major factor for *in utero* transmission of HIV^28–31^. In this study, maternal viral loads ranged from 1.7 log_10_ to 5.5 log_10_ copies/ml. Interestingly, expanded T cell clones were found in 1 of the 6 HEU infants born to mothers with the highest viral loads, suggesting that vertical transmission of HIV is influenced by several factors.

It is known that HIV is able to infect various tissues associated with pregnancy, including trophoblastic cells, Hofbauer cells, or placental macrophages^32,33^; and *in vitro* studies have shown that HIV is able to replicate in these tissues^34,35^. This results in a potential exposure of the fetus to HIV particles or HIV products over the course of pregnancy. Furthermore, HIV replication is known to create a pro-inflammatory environment within HIV-infected patients by continuously weakening mucosal barriers, leading to persistent translocation of microbial products^36^. This is true even in the presence of ART where systemic virus replication is undetectable. The pro-inflammatory environment is characterized by a variety of altered cytokine levels potentially affecting the development of the immune system of the fetus during pregnancy^6^.

Various socio-economic factors, such as household income or educational attainment, are difficult to control for and can contribute to the biasing of data to varying degrees. Thus, both HEU and HU cohorts were enrolled from similar communities in Nairobi at the same study site. We selected HEU and HU infants with similar growth and maternal STI history. These criteria led to only healthy infants of both groups being included in the study presented here. We did not find a correlation between the diversity measurement and the health status of the infants, monitored for up to 2 years after birth. In addition, this study could not identify a correlation between viral load and diversity scores in HEU infants. We predict a higher ratio of infants showing TCR clonotype expansion in HEU infants with increased morbidity.

An advantage of this study is the great similarity of the samples when it comes to other socio-economic factors or HIV treatment. A significant limitation in interpretation of most studies of HEU children is the influence of antiretroviral therapy, which mothers receive during pregnancy and/or infants receive in the weeks after birth. It is known that antiretroviral drugs are able to cross the placental barrier with different efficiencies depending on the specific drug, and that this perinatal drug exposure causes changes in blood composition including immunological relevant populations like T cells and neutrophils^17,37–40^. This confounding factor was excluded from this study by using ART treatment-naïve samples. In addition, the selection of mother-child sample pairs with identical health data at birth and similar epidemiological data within the first two years of life, such as data on infection types and number of infections, was aimed at eliminating further confounding factors. At the same time, the different sampling year may have led to minor differences between the HEU and HU group.

Technical factors, such as long-term storage or the HIV infection itself must be considered as confounding factors as well. While the HIV infection of mothers of HEU infants is an inherent characteristic of this sample group, the impact of the duration and conditions during long-term cryopreservation was assessed by measuring RIN scores. In fact, different RIN scores were measured between the samples of the HEU and HU infant groups, and samples of the HU group showed lower RIN scores than HEU samples. It is likely that the lower RIN scores of HU samples were caused by conditions during long-term storage or transport. However, we conclude that the lower RNA quality had a uniform effect across all TRB sequences, leading to lower reading depth in HU samples but did not introduce any bias regarding the diversity measurements. This conclusion was supported by the observation of similar TRBV expression patterns between HEU and HU cord blood samples and by comparison with other studies^22^. Additionally, our focus on clonotypes of higher rank based on frequency (top 200 analysis) ensured that an effect caused by RIN scores within the HU group was minimized.

Finally, it should be noted that the T cell receptor repertoire of most T cells is defined by the combination of T cell receptor alpha (TRA) and TRB chains. The determination of the T cell receptor repertoire based on TRB sequences thus has certain limitations. However, the analysis of the combination of TRA and TRB chains is only possible by using newer technologies which are based on single cells. At present, these technologies are still very costly and their use can only be justified by a significant gain in knowledge. For this study sequencing of TRB sequences alone was favored over the sequencing of TRA/TRB combinations, which remains to be a common method for determining T cell diversity.

Our data indicate that the adaptive immune repertoire of an unborn child can be shaped by maternal chronic HIV infection. We identified differences in the TRB repertoire diversity of healthy infants born to treatment naïve HIV-positive mothers, which may explain observed differences in morbidity and mortality among HEU infants. These immunological differences to unexposed uninfected infants might be even more pronounced in the context of infants with more severe infections within the first few years after birth or other epidemiological anomalies.

## Methods

### Samples

Samples from two previously conducted studies were used for the analysis performed in this publication. Samples of HEU infants were collected during a study carried out in Nairobi, Kenya between 1992 and 1997^20^. Intervention measures for PMTCT of HIV were not in place at the time of this study. Anticoagulated infant venous umbilical cord blood was collected at birth after clamping the cord in two places before venipuncture. The overall health status of newborns, as well as additional epidemiological and socio-economic data, were collected. Children’s HIV status was determined using HIV DNA PCR at regular intervals up to 2 years after birth.

Samples of HU infants were collected between 2003 and 2005^21^. Mothers enrolled in this study were negative for HIV using two HIV-1 rapid tests (Determine HIV 1/2 test, Abbott Laboratories, Abbott Park, IL; Unigold HIV-1 antibody test, Trinity Biotech, Bray, Ireland). As before, study participants were recruited at delivery, where cord blood and epidemiological and socio-economic information was collected. Cord blood mononuclear cells (CBMCs) from both studies were processed and subsequently cryopreserved in liquid nitrogen.

Selection criteria for samples from HEU infants were based on maternal HIV viral load during pregnancy, which was used as a surrogate for the level of fetal exposure to HIV. We selected mother-infant pairs based on a proportional representation of infants born to women within the full range of maternal viral load observed in the parent cohort study. Additionally, women included in this study had no history of sexually transmitted infections (STIs) nor received STI treatment at enrollment. Only vaginal births were included in this study and newborns were of a size (IQR 46–50 cm) and weight (IQR 2,900–3,500 g) appropriate to the norm. Selection criteria of HIV-unexposed (HU) samples had identical eligibility criteria in terms of STIs and birth characteristics.

Both original studies were reviewed and approved by the institutional review boards (IRB) of the University of Washington and the University of Nairobi, conducted within the guidelines of the Declaration of Helsinki, and all women gave written informed consent. The results of the current study reported here followed IRB regulations and guidance of the University of Washington and the University of Rhode Island, and were approved by the Kenyatta National Hospital-University of Nairobi joint Ethics and Research Committee.

### RNA/DNA extraction and cDNA synthesis

CBMC samples were thawed using RPMI-1640 (Fisher Scientific) +10% fetal bovine serum (Thermo Fisher) +50 U/mL Benzonase (EMD Millipore, 71205–3) and nucleic acids were extracted using the AllPrep DNA/RNA Mini Kit (Qiagen, Hilden, Germany). All DNA extraction steps were performed manually, while all RNA extraction steps following the DNA removal were performed using the automated QIACube (Qiagen, Hilden, Germany). RNA collection was performed in 30 *μ*L nuclease-free water. RNA concentration was determined using the NanoDrop. RNA quality assessments were carried out by determining RNA integrity numbers (RIN scores), which were determined using the Bioanalyzer instrument and RNA Nano kits (both Agilent Technologies, Santa Clara, US).

### Library preparation

The TRB chain sequence is defined by the complementarity determining region 3 (CDR3), a region that encompasses the variable (V), diversity (D), and joining (J) gene regions. Using an initial single-primer reverse transcriptase step, including a unique molecular identifier (UMI) sequence of 12 nucleotides length, 22 different TRB genes were targeted and amplified. Libraries compatible with Illumina’s MiSeq system were prepared using three consecutive reactions performed on the extracted RNA. First-strand cDNA synthesis was performed using a T cell receptor beta constant (TRBC) gene-specific primer together with SuperScript IV Reverse Transcriptase (Invitrogen) and purified using 1.0× Ampure XP beads (Beckman Coulter). Second-strand cDNA synthesis was performed using the high-fidelity Q5 Polymerase (NEB, Ipswitch, US), Betaine and a TRBV-specific primer pool followed by an additional purification step with 1.0× Ampure XP beads. Finally, full-length Illumina Nextra adapters were added to the double-stranded cDNA molecules using Q5 Polymerase together with a combination of Nextera primers containing indices for multiplexing different samples. Sample libraries were purified using Ampure XP beads and quantified using the dsDNA HS Qubit assay (LifeTechnologies). Size distribution analysis of sample libraries was performed using the Bioanalyzer and DNA 1000 kit (Agilent). Final library pools, which contained 12 sample libraries, were quantified by real-time PCR (KAPA Library Quantification Kit for Illumina platforms, KAPA Biosystems, Wilmington, US) and used in a final concentration of 4 nM within the Illumina denaturation and flow cell loading protocol (Illumina, San Diego, US).

### Next-generation sequencing

Amplicons of 22 TRBV families of 19 HEU and 9 HU cord blood samples were sequenced using Illumina’s MiSeq system. Sequencing was performed as 2 × 150 paired-end reads using MiSeq Reagent Kits v2 (Illumina, San Diego, US) with a 9% PhiX spike. Raw files were downloaded from Illumina’s BaseSpace repository for analysis.

### Data processing

Prior to sequence alignment and T cell receptor beta chain determination, quality scores of raw FASTQ files were assessed using the FastQC software. TCR-specific analysis steps, including UMI extraction and mapping of assembled consensus sequences to the International Immunogenetics (IMGT) database^41,42^, were performed using the MiGEC software^43^. The resulting tables, which contained the information on the identified TCR sequences, were further analyzed using R software^44^. The vegan and tcR packages were used to calculate the diversity scores^45,46^. For the visualization of the data, the ggplot2 package was used^47^.

### Diversity calculations: simpson, shannon, clonality, and rényi’s entropy

Based on the abundance of highly expressed T cell receptor beta (TRB) genes, several diversity scores were calculated for each sample. Since each mathematical characterization of diversity has different weightings and therefore varies in its variance and significance, it is generally accepted to determine a number of different diversity calculations and to evaluate them on the basis of their pattern. Shannon^48^ and Simpson^49^ scores are widely used in the field of ecology and have recently been applied to TCR and BCR diversity estimates. Shannon diversity represents a diversity measure with a weighting parameter of *α* = 1 and Simpson diversity a diversity measure of *α* = 2. When determining diversity scores, the following applies: The higher the value of the weighting parameter *α*, the greater the influence of high abundance T cell clones. Simpson diversity (*D*) and Shannon diversity (*H*, also known as Shannon-Wiener or Shannon-Weaver index) were used as a diversity measure for the TRB chains found in HEU and HU cord blood samples^50^. The formulas are given below, where *p*_*i*_ is the proportion of clonotype *i*, *S* is the number of clonotypes, and *b* is the base of the logarithm.

$$D=\sum {p}_{i}^{2}$$

$$H=-\,\sum {p}_{i}\,{\mathrm{log}}_{b}\,{p}_{i}$$

In addition to Simpson and Shannon diversity scores, clonality (1 - Pielou’s evenness, *J*) and Rényi’s entropy (*H*_*α*_) were calculated using the formulas below. Rényi’s entropy, which is related to Simpson and Shannon diversity scores, is given in the range of *α* = 1 to *α* = ∞, resulting in what is known as diversity profiles^51^. Clonality is based on Pielou’s evenness, which in turn is based on Shannon diversity score. Pielou’s evenness results in a value between 0 and 1. For clonality, the higher the value, the lower the evenness of the samples.

$$J=\frac{H}{\mathrm{log}\,S}$$

$${H}_{\alpha }=\frac{1}{1-\alpha }\times \,\mathrm{log}\,\sum {p}_{i}^{\alpha }$$

### Statistical testing

Non-parametric Wilcoxon rank-sum test was calculated for group mean diversity scores using R software^44^. Test results are displayed within the graphs together with significance levels. The final dataset was controlled for outliers using the univariant approach. Observations outside the range of 1.5× IQR were defined as outliers and removed from the dataset. Only one data point met these criteria and was removed from the dataset of the HU infant group.

## Data Availability

The datasets generated and analyzed during the current study are available from the corresponding author on reasonable request.
